# Book Reviews

**Published:** 1807-12

**Authors:** 


### CRITICAL ANALYSIS
### OF THE
### RECENT PUBLICATIONS
### ON THE
### DIFFERENT BRANCHES OF PHYSIC, SURGERY, AND MEDICAL PHILOSOPHY.

---

*Chemical Philosophy, or the eſtabliſhed Baſes of Modern Chemiſtry. Intended to ſerve as an elementary Work of that Science.* By A. F. FOURCROY, Councellor of State, Member of the National Inſtitute, one of the Commandants of the Legion of Honour, and Profeſſor of Chemiſtry. 3d Edition, conſiderably enlarged and amended. Tranſlated from the French. By W. DESMOND, Eſq. 8vo. P. 290. Symonds, 1807.

IT cannot be diſputed, that the French greatly excel all other nations in the ſimplicity with which they deſcribe the inſtitutes of a ſcience. Whether this ariſes from the frequency with the *ſcavans*, converſe with the ladies, and by that means are accuſtomed to revert ſo perpetually to firſt principles, we pretend not to aſcertain. But certain it is, that our beſt books of inſtitutes have uſually been tranſlations from that language. It muſt, at the ſame time, be admitted, and probably from the ſame habit of converſing often with thoſe who are entirely ignorant of the ſubject, that in too many inſtances, they have either given plauſible explanations of what was not underſtood, or elſe have paſſed over thoſe ſubjects altogether. From the known character of the celebrated author of the preſent work, we could not apprehend any danger of this kind, and from his national habits, we might expect thoſe advantages which we have been accuſtomed to meet with in his countrymen. In both inſtances it is unneceſſary to add, we have been amply ſatisfied. The work has already appeared before the public in two former editions, and the third differs from them in nothing but in keeping pace with every improvement which Chemiſtry has ſince received.

Another very great advantage attending this edition, is the introduction troduction. The former editions were rather intended for thofe readers who had already attained fufficient practical knowledge of Chemiftry in the old fchool, but were unacquainted with thofe principles on which the art now refts. In the prefent, the author attempts to inftruct thofe who are altogether uninformed, and in this he affumes the fame eafy familiarity of language, as in the reft of his work. In all he is equally fuccefsful; but we fhall more particularly direct our attention to the introduction, as no part of it has hitherto appeared before the public.

"The efpecial object of chemical philofophy being, 1ft. To apply the general theory of chemiftry to the phenomena of nature and to the operations of art, the caufe and effects of which are entirely within the province of this fcience; 2nd. To fhew the connections exifting between thefe phenomena and their reciprocal influence upon each other, we muft confider this philofophy as comprifing the whole of the important difcoveries made by chemiftry.

"But, in order to conceive the whole of thefe great truths of chemiftry, to perceive their relations and connections, to underftand clearly the expreffions or propofitions employed to defcribe them, particularly if we fuppofe that thofe who wifh to become mafters of them, apply, for the firft time, to the ftudy of chemiftry, it is indifpenfably neceffary that they fhould be preceded by a clear explanation of the firft principles of the fcience, or of the elementary notions on which its bafis refts. This explanation is the fubject of the prefent introduction.

"From the flighteft obfervation of what takes place around us, between the various productions and powers of nature, we perceive that beings inceffantly vary, and never continue in a permanent ftate: even the moft folid bodies, and apparently the moft unalterable, fuch as the maffes and fummits of mountains, the hard rocks and ftones which form them, are fplit, fly off, are divided and reduced by the progreffive effects of the fun, of rains, of cold, of heat. The *ftrata* or layers of the earth, penetrated by water, opened and expofed to the air, undergo continual alterations by the prefence or abfence of the waters, by the atmofphere, meteors, &c. Vegetals and animals are affected by thefe external influences, and undergo perpetual changes; they abforb, by their pores and veffels, liquids and elaftic fluids, which they appropriate to themfelves, convert them into their own fubftance: thefe fluids add to their weight, promote their growth, and preferve their life, or threaten them in a thoufand different ways, by augmenting or diminifhing their vital powers.

"Thefe alterations of ftones, of minerals, and of foffils; thefe functions of vegetals and animals, either fupported or difturbed; thefe changes, of whatever defcription, introduced into all the productions of nature, by their reciprocal action, when they depend upon any other properties than their form, their extenfion, their confiftence, &c. when they are connected with internal actions, which take place between the minute particles of bodies, belong entirely to chemical powers, and conftitute the proper object of the ftudy of chemiftry.

"This

"This confideration fhews the neceffity of defining this fcience, and of diftinguifhing it from all other natural fciences.

"For the purpofe of conceiving and explaining the very numerous changes juft mentioned, chemiftry however employs but two general methods, by which it examines and fucceeds in imitating, on a fmall fcale, the operations of nature; it is enabled, by thefe means, to determine the kinds and differences of thofe bodies one from another; and the order of their compofition. The chemift foon difcovers, by the means which he employs, that all the phenomena, which he obferves or produces, depend on a power placed by nature in the conftituent molecles of bodies; whence he is led to ftudy the laws of this power, and thus fucceeds in re-producing, upon a fmall fcale, the effects which he obferves in nature itfelf.

"As he proceeds in this ftudy, he afcertains the neceffity and poffibility of claffing natural bodies, of accurately comparing them with each other, and of regularly dividing them.

"By determining the advantages and extent of the fcience, the chemift foon attains to the explanation of the phenomena of nature. He conceives the caufes and the effects of thefe phenomena, and, what is ftill more fublime, he is at length enabled to compare them with each other, to point out their mutual dependence, their connections, their relations, and their claffification; fuch is, as we fhall clearly demonftrate at the end of this introduction, the true aim of the Chemical Philofophy."

Highly as we refpect the Herculean abilities of Fourcroy, we cannot help wifhing that he had avoided falling into the errors of moft of his brethren. It is very true that the "alterations of ftones, minerals, and foffils, the changes of whatever defcription introduced into fuch productions when they depend on any other action than their form and extenfion, confiftence, and other fimilar properties; when they are connected with internal actions, which take place between the minute particles of bodies, belong entirely to chemiftry." In a certain degree alfo, the fame may be faid of animals and vegetables. But, "the fupport and difturbance of their functions" is connected with thofe powers, and thofe actions which only exift during life. It is, therefore, of the utmoft importance, to feparate thefe proceffes from thofe chemical changes which we can imitate in a certain degree, but not in fuch a degree as to fupport, much lefs to renew, or begin thofe functions. Though life is fupported by the infpiration of atmofpheric air, and though we can imitate that change which the blood affumes by the application of that fluid, yet we can neither apportion the quantity, nor can we, under every circumftance, excite thofe actions by which only its application can be ufeful to living animal, or vegetable bodies. All our author can mean is, that we can demonftrate, however various may be the appearance of animated nature, yet that the whole is reduceable to certain elements, the attractions of which we can explain, by fhowing them in inanimate matter, but cannot pretend to imitate, inafmuch as they are governed by laws, which human power can never expect to attain. We are aware it will be faid, that no no one will accufe fuch a philofopher of attempting to animate clay, or even to refufcitate an animal, whofe organic ftructure may feem to remain entire, but in whom the power of action is for ever loft. Such an accufation is far from our meaning; but when a work is intended for the inftruction of the mere learner, every poffible ambiguity fhould be avoided. The prefent paffage might be much improved, if the fentence concerning animals and vegetables were omitted, and a feparate paragraph devoted to thofe fubjects in which a diftinction fhould be made between the living and dead ftate.

The reft of the introduction, contains "the general means of chemiftry," that is, the ftructure and furniture of the elaboratory; "the nature and principles of bodies," (terms very properly fubftituted by our author for the *elements* of the Ariftotelians;) the attraction of aggregation," or the power by which homogeneous bodies retain their figures, whether folid, liquid, or elaftic vapours; "the attraction of compofition," that is, when two bodies have that attraction for each other, that when powdered, however finely each particle or grain, confifts of the fame proportion of each fubftance, as whilft the mafs remained entire. *Particles* is the name given to the fubftance thus divided. When the bodies are feparated, and afterwards pulverized, the name of *conftituent molecules* is given to the powder. To illuftrate this, cinnabar when powdered, is faid to confift of *particles*. When the mercury is feparated from the fulphur, and each of them pulverized, the powder is named *conftituent molecules*.

A general view of *chemical operations* follows, as performed by means of fire, of water, and of other liquids; the claffification of bodies; the phenomena of nature and art; their claffification conftituting the chemical philofophy, make up the remainder of the introduction.

The body of the work is formed on the models of the former editions, only altered as the fcience has been further improved. We fhall extract the defcription of blood, as it may ferve to illuftrate further the neceffity of that diftinction, which we regretted to find omitted in the introduction.

"*Blood:* a red fluid, warm at thirty degrees [Reaumur] in man, quadrupeds, birds; at the temperature of the medium which they inhabit, in oviparous quadrupeds, ferpents, fifhes; filling the arteries and veins; proceeding through the former from the heart to the extremities and thence returning to the heart by the veins; by this motion conftituting circulation; a liquid fomewhat vifcous, fweetifh, concrefcible by cold, mifcible with water; dividing itfelf almoft fpontaneoufly into three different fubftances, *white ferum, red ferum*, or the colouring part, and the *fibrous matter*.—Each of thofe matters poffeffes diftinctive characters, namely: the alkalinity of the ferum; its coagulability by fire, by metallic oxyds, &c. (a coagulability arifing from a more intimate combination of oxygen and very fimilar to that of the white of an egg, from which refemblance ferum has been denominated an albuminous liquor); the fame is the general general nature of the red ferum, which differs from the white only by the prefence of the phofphat of iron highly oxyded; the fpontaneous concreffibility of the fibrous matter (fibrin); its diffolubility in weak acids, its property of yielding much ammoniac and oil by diftillation.

"These principal characters are to be confidered in the mafs of the blood, which appears to be the primitive principle of all animal fubftances and the common origin of all the humours and all the folids. It has been named flowing flefh, on account of the fibrin which becomes concrete in it by cooling. The caufe of its heat has been determined to confift in the alteration and abforption of vital air by refpiration. The renewal of the blood has likewife been determined to be performed by the chyle, and the converfion of the latter into animal matter, by the difengagement of a great quantity of carbon and hydrogen, which difengagement appears to take place in the lungs. It carries warmth, motion, and nourifhment to all the parts: it is a truly vital fluid."

The only objection to this paffage is, that blood is faid to divide itfelf *almoft fpontaneoufly*, into three different fubftances. The two words *almoft fpontaneoufly*, are extremely unphilofophical. Had the firft been omitted, the laft might have been faid to be logically correct, inafmuch as the author admits the vitality of blood, (and life) implies a fpontaniety of action. But this is very different from a chemical change, and the term is rendered defultory by the addition of *almoft*. It may be added that, if blood lofes its life before its feparation, fuch feparation cannot be accomplifhed but by other mixtures. The paffage fhould therefore have been "dividing itfelf under cirtain circumftances in the body, and always if it retains its life out of the body, into three different fubftances."—

The tranflation is for the moft part correct, though not without Gallicifm. In one inftance the tranflator has been fo kind as to reform our language. The readers will perceive, in the different extracts, the word *vegetal* for vegetable. This we are told is more correct, and certainly it is fo, nor do we object to the alteration, though at firft it muft offend our ears. As, however, there is little reafon to hope that the change will be generally adopted, we think it was fcarcely worth while to begin it. The other remarks on the impropriety of adopting the French terminations, are very judicious.

It is unneceffary to add any thing in commendation of a work, the neceffity of which is fo obvious, and the writer fo univerfally efteemed.

---

### *The Edinburgh Medical and Surgical Journal, No. XII.*

[ Concluded from our last, pp. 468—476. ]

Article 8.—*Dissection of Two Cases, in which a moveable Body was found in the Cavity of the Vaginal Coat of the Testis, illustrated by an Engraving: with Remarks.* By JAMES WARDROP, Fellow of the Royal College of Surgeons, at Edinburgh.

Article 9.—

Article 9.—*Case of Gout in an African Negro.* By ANDREW DUNCAN, Senior Professor of the Institutes of Medicine in the University of Edinburgh.

THIS paper, though somewhat tedious, contains many useful practical remarks. That the disease was gout, will, we presume, be disputed by no one, unless such as having given a contrary opinion during the patient's life, may chuse to retain it. But, the principal advantage of the paper, is to show the absurdity of those undefined terms of *retrocedent and misplaced* gout. By the dissection, it is evident that all the parts affected were inflamed, and no one who sees gout in the toes, will doubt inflammation there also. It is therefore much to be regretted, that in gouty subjects, we should be so much afraid of evacuants of every kind. When gout, as it is called, attacks the head, that is, when inflammation leaves the toes and seizes the head, we have the authority of Sydenham and his copyers to bleed. Sydenham went further, and advised that young plethoric subjects should be bled early in the disease.—His successors have attached so much the idea of local plethora to general debility, as to be fearful of any thing more than topical bleedings; we are ready to admit, that even local bleeding may be too much in subjects debilitated by previous sufferings, but more powerful and general evacuations may be safely used in those cases, in which it is recommended by the great master above-mentioned.

Article 10.—*Observation on the History and Treatment of an Epidemic Ophthalmia, which appeared in the* 4th *Battalion of Royals in Edinburgh Castle, during the Months of July and August,* 1807. By C. F. FORBES, Esq. Surgeon of the Royals.

SOME curious particulars are added to the history of the ophthalmia in his paper.—The inflammation was always confined to the conjunctiva. In three fourths of the cases, the right eye was attacked. Some times in the course of an hour the other eye became affected, but more commonly whilst the first was recovering. In no instance did the inflammation return to the eye first affected. In these, as in some other accounts of the complaints, no uneasiness was felt from the free admission of light. If suppuration took place, it was always in the conjunctiva, from which, in some instances, the matter found its way through the palpebra.

In the cure, the author found that the bold antiphlogistic plan to which only the disease has in other instances yielded, was altogether unnecessary. A low diet, keeping the bowels open, and the application of various topical remedies, were sufficient. Scarification of the eye was found very serviceable, and particular advantage derived from the use of an instrument invented by Dr. Wardrop.

" It consists of a straight blade, about one fifth of an inch broad, with parallel sides, and an obtuse slightly rounded cutting extremity. With an instrument of this form a number of vessels can can be divided by drawing it slightly in different directions along the internal surface of the eye-lid, very readily, and without the least risk of making any deep puncture, or of wounding the eye-ball."

This description should have been more detailed, or illustrated with a plate.

A long paper of the "Inquirer" closes this division of the Number. It is entitled "On some convulsive diseases common in certain parts of Scotland." These are principally extracted from Sir John Sinclair's Statistical Account. They show nothing more than what is universally admitted, that the influence of the imagination over the bodily organs, is at times so considerable, as to confound all symptoms, and that the general sympathy on those occasions, is great in proportion, as mankind are secluded from general intercourse, particularly when religious enthusiasm becomes the prevailing fancy.

